# Evaluation of Physicochemical and Cooking Characteristics of Rice (*Oryza sativa* L.) Landraces of Lamjung and Tanahun Districts, Nepal

**DOI:** 10.1155/2020/1589150

**Published:** 2020-08-18

**Authors:** Amrit Pokhrel, Anup Dhakal, Shishir Sharma, Ankur Poudel

**Affiliations:** Department of Agronomy, Plant Breeding and Agri-statistics, Institute of Agriculture and Animal Sciences, Lamjung Campus, Tribhuvan University, Nepal

## Abstract

After the Green Revolution, the increase in the choice of modern varieties at the expense of landraces has become a major cause of varietal loss. The preference, choice, and the economy of rice (*Oryza sativa* L.) largely depend on its physicochemical and cooking properties, which are found to be superior for landraces than modern varieties. In this study, we assessed and evaluated milled rice of 30 rice landraces on their physicochemical and cooking characteristics which aim to promote the revival of old landraces. Six parameters of physical properties, four parameters of chemical properties, and five parameters of cooking properties were evaluated based on the standard protocols. Significant variations (*p* < 0.05) were found in all the properties that were evaluated. The result showed that the highest milling recovery was found in Indrabeli (75.55%) whereas the lowest was found in Kalo Masino (66.98%) and bulk density ranged from 0.81 g/cm^3^ to 0.88 g/cm^3^ showing not much variability. Although most of them were of medium grain type, their 1000 kernel weight varied between 12.62 g and 25.65 g. From the observed chemical properties, Pahelo Anadi (9.73 ± 0.55 mm) showed the highest gel consistency and lowest apparent amylose content (7.23 ± 0.36%). Also, 13% of landraces possessed strong aroma while noble cooking properties were showed by Thakali Lahare Marsi with the highest elongation ratio (2.41 ± 0.05) and by Chiniya with the lowest gruel solid loss (0.033 ± 0.03%) and minimum optimum cooking time (23.45 ± 0.03 min). In the principal component analysis, the first four principal components retained 73.8% of the variance. The first and second principal components were mostly related with the physical and chemical characteristics while the third and fourth principal components were concerned with cooking characters. Superior characters possessed by rice landraces can be further assessed for the breeding programs so that the cultivation of these cherished rice landraces can be enhanced.

## 1. Introduction

Rice (*Oryza sativa* L.) has occupied the position as one of the leading food crops of the world that feeds nearly over one-half of the world population as a staple food [1]. Rice is widely cultivated throughout the world and is the second most important cereal crop in terms of cultivation after wheat [2]. It is a member of the Poaceae family and its domestication started from ancient civilisation native to Southeast Asia. According to Bandumula [3], approximately 90% of the rice is grown and consumed in Asia and eleven Asian countries contribute 87% of global rice production. Rice is mainly consumed as a whole grain after cooking which contributes about 40% to 80% of the calorie intake [4]. The rice landraces have been grown and consumed since time immemorial and have an important role in livelihood and food security [5]. The rice landraces have varied agro-morphological characters, and some of the landraces are promising in terms of yield [6]. According to Hegde, Yenagi, and Kasturiba [7], the traditional landraces have been used for its higher nutritive value than the hybrid and also have been used in Ayurveda system since ancient times in order to treat different diseases like diarrhoea, fever, vomiting, haemorrhage, burns, improve eyesight, vocal clarity, fertility, and many more. These health benefits lead to their increasing demand towards consumers. These days, there is increasing demand for rice varieties with excellent quality characteristics throughout the world since cooking and eating quality have the major role in determining economy, market, and consumer acceptability [8].

Although rice hybrids are known to show a very high level of heterosis for grain yield, they suffer a huge problem in grain quality mainly because of the high degree of grain quality diversity of parental lines [9]. With the consumers being more concerned for the quality, emphasis on quality breeding has assumed a greater significance in recent years. The grain quality of rice is a complex character composed of many components such as nutritional quality, appearance, cooking quality, and eating quality. A wide variation in the chemical composition of rice landraces provides further scope for improving its nutrition and grain quality through breeding. The information on the physicochemical and cooking qualities of rice landraces under investigation is not available or still limited in Nepal. Keeping these facts, the present investigation was undertaken with the objectives to characterise and evaluate physicochemical & cooking characteristics along with assessment of grain quality of landraces for selection and further breeding programmes.

## 2. Materials and Methods

The landraces used in this research were collected from Marsyangdi-Chepe Community Seed Bank, Rainas Lamjung, Nepal; local farmers of Rainas Lamjung; and Purkot Community Seed Bank, Bhanu Tanahun, Nepal; and then grown in the Agronomy farm of Institute of Agriculture and Animal Science (IAAS), Lamjung Campus, in irrigated transplanting condition during July-November, 2018. Rice was harvested manually, threshed, cleaned, and the moisture percentage was decreased to 12%. The samples were then shelled using the Otake Rice Sheller (Impeller husker, FCS type, Otake Co., Oharu, Japan) in order to obtain the brown rice. The obtained brown rice was polished in Yamamoto Rice Polisher (Friction type, VP-31T, Yamamoto Co., Tendou, Japan) to obtain milled rice of 4% degree of milling. Shelling and polishing activities were done in the Food Research Division, Nepal Agricultural Research Council, Khumaltar, Lalitpur. All the lab experiments were carried out in the Laboratory of IAAS, Lamjung Campus, in February 2019.

### 2.1. Milling Characteristics of Rice

#### 2.1.1. Dehusking

The obtained brown rice after shelling was weighed to determine the hulling percentage with the equation given by Bhattacharya [10].

(1) $$\begin{array}{c} \text{Hulling percentage}=\frac{\text{Weight of brown rice }\left(\text{g}\right)}{\text{Weight of rough rice }\left(\text{g}\right)}*100\% \end{array}$$

#### 2.1.2. Polishing

The obtained milled rice after polishing was weighed to determine the milling recovery of the rice sample using the following equation given by Shaeed, Akinoso, and Nahemiah [11]; Bhattacharya [10]:

(2) $$\begin{array}{c} \text{Milling recovery}=\frac{\text{Weight of milled rice }\left(\text{g}\right)}{\text{Weight of sample paddy used }\left(\text{g}\right)\;}*100\% \end{array}$$

### 2.2. Physical Characteristics of Rice

#### 2.2.1. Bulk Density (g/cm^3^)

The bulk density (g/cm^3^) of milled rice was calculated according to the equation used by Fraser, Verma, and Muir [12]:

(3) $$\begin{array}{c} \text{Bulk density }\left(\rho \text{b}\right)=\frac{\text{Mass of rice }\left(\text{g}\right)}{\text{Volume of vessel }\left(100\text{ml}\right)} \end{array}$$

#### 2.2.2. Grain Length and Breadth (mm)

As per the method mentioned by Nadvornikova et al. [13] and Shaeed, Akinoso, and Nahemiah [11], for grain dimension, thirty rice kernels were randomly selected for measurement for their length (mm) and breadth (mm) using a digital Vernier caliper with least count of 0.01 mm. On the basis of their length, IRRI further classified rice cultivars as extra-long (>7.5 mm), long (6.61-7.5 mm), medium (5.51-6.60 mm), and short (<5.5 mm) [10].

#### 2.2.3. Grain Shape

Grain shape here is referred to as the length-width ratio, which is calculated using the equation

(4) $$\begin{array}{c} \frac{L}{W}=\frac{L\;\left(\text{mm}\right)}{W\;\left(\text{mm}\right)} \end{array}$$

where *L*/*W* is the length-width ratio of milled rice, *L* is the average length of milled rice (mm), and *W* is the average width of milled rice (mm). Based on the length-width ratio, grains were classified according to the classification provided by IRRI: slender (>3.0), medium (2.1-3.0), bold (1.1-2.0), and round (<1.1) grain types [10].

#### 2.2.4. 1000 Kernel Weight (g)

This was determined by counting 1000 kernels from each sample randomly and weighing 1000 kernels of each landraces separately [14].

### 2.3. Chemical Characteristics of Rice

#### 2.3.1. Gelatinization Temperature by Alkali Spreading Value

For the alkali spreading test, seven whole kernels of each landraces were taken in Petri dish with three replications; then, 20 ml of 1.7% potassium hydroxide (KOH), 1.7 g in 100 ml of distilled water, was added to the sample and kept undisturbed for 23 hours at room temperature of 21°C. The spreading value of each replication was rated visually on a 7-point numerical scale. Average scores of seven kernels were taken as the spreading value [15].

#### 2.3.2. Gel Consistency (GC)

The gel consistency was determined following the method given by Cagampang, Perez, and Juliano [16]

#### 2.3.3. Apparent Amylose Content (AAC)

For amylose content determination, the method given by Juliano [17] was used.

#### 2.3.4. Aroma

200 mg (0.2 g) of whole grain milled rice kernel was weighed and placed in a culture tube. 0.5 ml of 0.1 N KOH was pipetted into the culture tube and closed with the lid. After 15 min, the lid was opened and smelled. Three persons were used as judges, and the mean score was taken. In case of doubt, the lid was again closed and was smelled again after 15 min [10].

### 2.4. Cooking Characteristics of Rice

#### 2.4.1. Optimum Cooking Time (OCT)

It was determined using the Ranghino method, where milled rice samples 2 g were placed in a 100 ml beaker and cooked in boiling hot water bath at 95^o^C in 20 ml of water. The optimum cooking time was estimated by pressing the cooked rice samples between two glass slides at regular intervals till no white starch core was left [18].

#### 2.4.2. Water Uptake Ratio (WUR)

According to Bhattacharya and Sowbhagya [19], rice samples of 2 g in 20 ml of water were cooked in a boiling water bath at 95°C for optimum cooking time. The water left was drained, and the cooked rice was transferred to a filter paper to absorb superficial water. The cooked samples were weighed accurately to calculate the water uptake ratio (WUR):

(5) $$\begin{array}{c} \text{Water uptake ratio}=\frac{\text{Weight of kernel after cooking }\left(\text{g}\right)}{\text{Weight of kernel before cooking }\left(\text{g}\right)} \end{array}$$

#### 2.4.3. Gruel Solid Loss (GSL)

Remaining residual water from the sample used for the water uptake ratio was used for the determination of the gruel solid loss. The weight of the empty Petri dish was measured and noted as *W*_1_ (g). Then, the remaining gruel liquor was poured in the Petri dish with few washings and the weight of the empty Petri dish with gruel was measured and recorded as *W*_2_ (g). The Petri dish was subjected to 110°C at a hot air oven for 5 hours, until the dry gruel was obtained. The weight of the Petri dish with dry gruel was measured and recorded as *W*_3_ (g) [2]. This procedure was done in triplicate for each treatment. The solid in gruel loss was calculated as *W*_3_–*W*_1_, where *W*_1_ is the weight of the empty Petri dish, *W*_3_ is the weight of the empty dishes+dry gruel [5]. The gruel solid loss was expressed in percentage as

(6) $$\begin{array}{c} \text{Gruel solid loss}\%=\frac{W_{3}–W_{1}\;\left(\text{g}\right)}{W_{2}-W_{1}\;\left(\text{g}\right)}*100\% \end{array}$$

#### 2.4.4. Elongation Ratio (ER)

The elongation ratio is given by the average length of cooked rice divided by the average length of uncooked rice [2]. The elongation ratio is expressed as

(7) $$\begin{array}{c} \text{Elongation ratio}=\frac{\text{Average length of cooked rice }\left(\text{mm}\right)}{\text{Average length of uncooked rice }\left(\text{mm}\right)} \end{array}$$

#### 2.4.5. Cooked Length-Breadth Ratio (CLBR)

The cooked rice length-breadth ratio was expressed using the equation [2]:

(8) $$\begin{array}{c} \text{Cooked length}­\text{breadth ratio}=\frac{\text{Length of cooked rice }\left(\text{mm}\right)}{\text{Breadth of cooked rice }\left(\text{mm}\right)} \end{array}$$

### 2.5. Statistical Analysis

The experiment was conducted under laboratory condition with three replications. The data obtained from the experiment were entered and recorded in Microsoft Excel. Analysis of variance (ANOVA), post hoc test using LSD, principle component analysis (PCA), and graphs were made using R and its packages-agricolae, FactoMineR, factoextra, and ggplot2.

## 3. Results

### 3.1. Milling Characteristics

#### 3.1.1. Hulling Percentage (HP)

The hulling percentage ranged from 76.18% to 82.53%, with CV of only 2.18%. Marsi had the highest hulling percentage of 82.53%, whereas Pahelo Anadi had the lowest hulling percentage of 76.18% followed by Kalo Masino (76.19%) (see Table 2 in the Supplementary Material for tabular representation).

#### 3.1.2. Milling Recovery (MR)

Milled polished rice obtained from whole grain varied from 66.98% to 75.55% among the landraces with a CV of 3.32%. The highest milling recovery was obtained in Indrabeli (75.55%) followed by Baryang Masino (74.93%), and the lowest milling recovery was obtained in Kalo Masino (66.98%) followed by Aanga (67.86%) (see Table 2 in the Supplementary Material for tabular representation).

### 3.2. Physical Characteristics

#### 3.2.1. Bulk Density

The maximum bulk density was obtained in Pudke Dhan (0.88 g/cm^3^) and Thakali Lahare Marsi (0.88 g/cm^3^) whereas the minimum bulk density was obtained in Mansara (0.81 g/cm^3^) and Kalo Masino (0.81 g/cm^3^). No wide variation was observed in bulk density as the value only varied between 0.81 g/cm^3^ and 0.88 g/cm^3^ with a CV of 2.34% (see Table 2 in the Supplementary Material for tabular representation).

#### 3.2.2. 1000 Kernel Weight

The thousand kernel weight for the landraces varied from 25.65 g to 12.62 g. Rato Masino had the maximum 1000 kernel weight of 25.65 g, whereas the minimum 1000 kernel weight was of Kalo Masino (12.62 g) (see Table 2 in the Supplementary Material for tabular representation).

#### 3.2.3. Grain Length and Breadth and Grain Dimension

**Figure 1:**
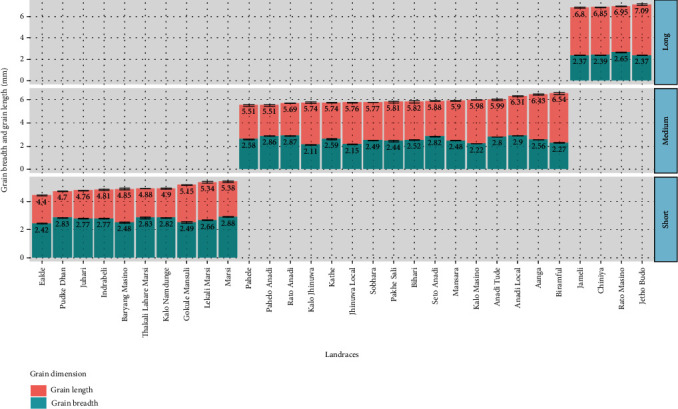
Classification of the landraces based on their grain dimension. In the graph, the grain length (red color) and grain breadth (green color) of the landraces are represented. The error bars represent the standard error of the mean (SEM) of measurements for 30 landraces in three replications.

As shown in Figure 1, three types of grain length were obtained- 4 long types, 16 medium types, and 10 short types. Both grain length and breadth were highly significant among the landraces (*p* value < 0.001). In long-type grain, the maximum length was seen in Jetho Budo (7.09 ± 0.084 mm) and the minimum length in Jarneli (6.8 ± 0.056 mm), in medium-type grain the maximum length was obtained in Biramful (6.54 ± 0.092 mm) and the minimum length in Pahelo Anadi (5.51 ± 0.069 mm), and in short-type grains the length ranged from Eakle (4.4 ± 0.041 mm) to Marsi (5.38 ± 0.059 mm). Although the grain breadth was significant between the landraces, their value only ranged from Kalo Jhinuwa (2.11 ± 0.021 mm) to Anadi Local (2.9 ± 0.003 mm) which suggest that variation in breadth among the landraces was not obtained as likely as the variation in length.

#### 3.2.4. Length-Breadth Ratio (LBR) and Grain Shape

The shape of the rice grain was expressed on the basis of length and breadth ratio. Significant difference (*p* < 0.001) was observed for the length-breadth ratio between the landraces. The length-breadth ratio was maximum for Jetho Budo (2.99 ± 0.035 mm) and minimum for Thakali Lahare Marsi (1.7 ± 0.028 mm) followed by Pudke Dhan (1.66 ± 0.02 mm) and Juhari (1.72 ± 0.012 mm). From the length-breadth ratio, landraces were further grouped based on grain shape, in which 11 were bold grains and 19 were medium-type grains.

### 3.3. Chemical Characteristics

#### 3.3.1. Gelatinisation Temperature

Among the landraces, 53% were found to have an intermediate gelatinisation temperature, i.e., of 70-74°C and 47% were found to have a high gelatinisation temperature, i.e., >74°C. However, no landraces were found with a low gelatinisation temperature, i.e., <70°C.

#### 3.3.2. Gel Consistency (GC)

The landraces were found significantly different for their average gel consistency (*p* < 0.001) (Table 1). Pahelo Anadi (9.73 ± 0.549 mm), Seto Anadi (9.27 ± 0.733 mm), and Anadi Tude (9.2 ± 0.3 mm) were found to be with the highest gel length, and Kalo Masino (1.27 ± 0.12 mm) was found to be with the minimum gel length. Great variation was observed among the landraces in gel consistency (CV = 37.36%), the landraces were further categorized based on their gel length as hard (12 landraces), intermediate (10 landraces), and soft (8 landraces).

#### 3.3.3. Apparent Amylose Content (AAC)

**Figure 2:**
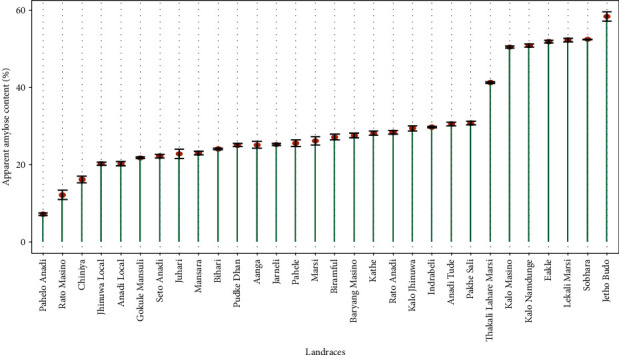
Plot of amylose content of landraces. The error bars represent the standard error of the mean (SEM) of measurements for 30 landraces in three replications.

Apparent amylose content was found maximum in Jetho Budo (58.39 ± 1.17%) and minimum in Pahelo Anadi (7.23 ± 0.365%). The landraces evaluated were found significantly different in their amylose content (*p* < 0.001), but only small variation was found among them (CV = 3.63%), as shown in Figure 2.

#### 3.3.4. Aroma

**Figure 3:**
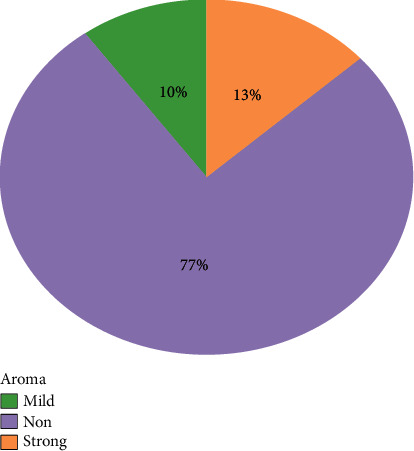
Percentage distribution of aromatic landraces-mild aroma (green color), no aroma (purple color), and strong aroma (yellow color).

As shown in Figure 3, almost 77% of the landraces were non scented, while the remaining 13% were strong scented and 10% were mild scented. Strong scented landraces were Biramful, Kalo Jhinuwa, Pahelo Anadi, and Rato Anadi, and mild scented landraces were Anadi Local, Kalo Masino, and Pahele.

### 3.4. Cooking Characteristics

#### 3.4.1. Optimum Cooking Time (OCT)

The results of the optimum cooking time for the rice varieties are presented in Table 1. The average cooking time of the landraces was found to be significantly different (*p* < 0.001). Cooking time was found to be maximum for Aanga (44.4 ± 1.464 min) followed by Pahele (41.40 ± 1.892 min) whereas minimum cooking time was found in Chiniya (23.45 ± 0.029 min) followed by Jarneli (23.85 ± 0.267 min).

#### 3.4.2. Water Uptake Ratio (WUR)

The water uptake ratio varied from 7.96 to 3.91 (Table 1), in which the highest was obtained from Jetho Budo (7.96 ± 0.037) followed by Biramful (7.52 ± 0.335) and the lowest was obtained from Rato Anadi (3.91 ± 0.032). A significant difference was obtained among the landraces for water uptake ratio (*p* < 0.001).

#### 3.4.3. Gruel Solid Loss (GSL)

The gruel solid loss in different rice landraces varied significantly. As shown in Table 1, higher solid loss was obtained in Rato Masino (2.84 ± 1.672%) followed by Jetho Budo (2.13 ± 0.144%) and Kalo Masino (1.82 ± 0.231%) whereas lower solid loss was obtained in Chiniya with just 0.33 ± 0.000% which can be regarded as a great characteristic. Greater variation was obtained among the landraces for gruel solid loss with CV equals 63.19%.

#### 3.4.4. Elongation Ratio (ER)

Thakali Lahare Marsi showed the highest elongation ratio (2.41 ± 0.053) which was significant than other landraces whereas the minimum elongation ratio was obtained in Rato Masino (1.20 ± 0.028) followed by Pakhe Sali (1.23 ± 0.024) and Kalo Jhinuwa (1.30 ± 0.052). The average elongation ratio of landraces varied significantly (*p* < 0.001); however, low variability was obtained among them (CV = 4.15%) (Table 1).

#### 3.4.5. Cooked Length-Breadth Ratio (CLBR)

The average cooked length-breadth ratio was significantly different among the landraces (*p* < 0.001) with the highest in Thakali Lahare Marsi (3.87 ± 0.065) followed by Jetho Budo (3.78 ± 0.09) and lowest in Kathe (2.05 ± 0.05) followed by Pakhe Sali (2.07 ± 0.062) (Table 1). In the cooked length-breadth ratiolow variability (CV = 5.97%) among the landraces was obtained.

#### 3.4.6. Principal Component Analysis

**Figure 4:**
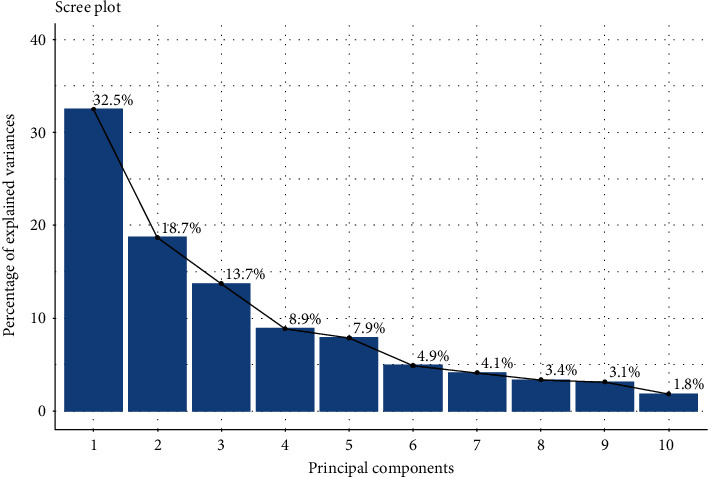
Contribution of each principal component to total variance.

**Figure 5:**
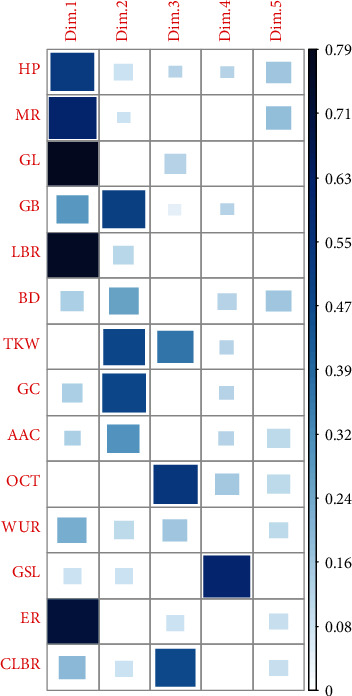
Principal components and their relation with variables.

**Figure 6:**
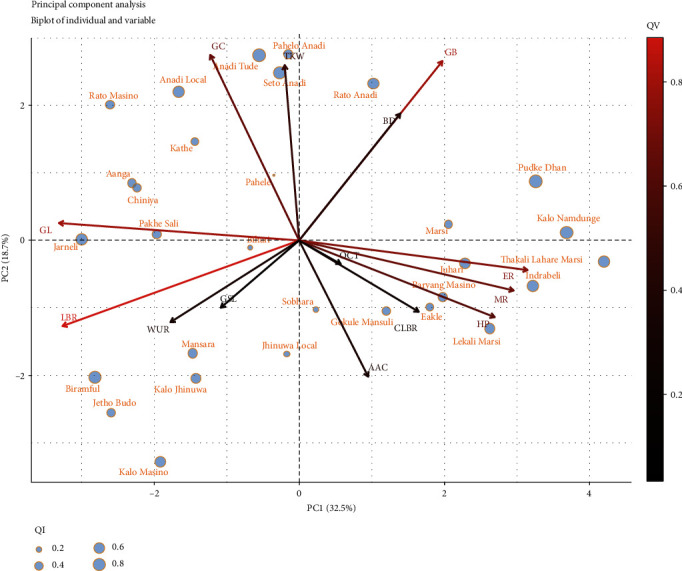
Projection of variables and individuals as of PC1 and PC2 on the factor plane for 30 rice landraces. QI is the quality of representation of individuals, and QV is the quality of representation of variables.

**Figure 7:**
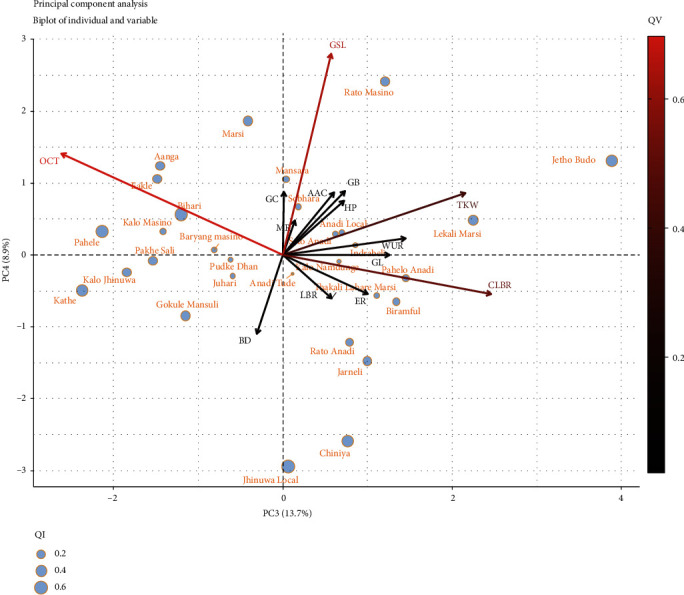
Projection of variables and individuals as PC3 and PC4 on the factor plane for 30 rice landraces. QI is the quality of representation of individuals, and QV is the quality of representation of variables. Note: BD=bulk density, TKW=thousand kernel weight, GL=grain length, and GB=grain breadth; other abbreviations are mentioned in Materials and Methods.

In this study, the principal component analysis (PCA) was carried out to summarize the information contained in the multivariate data (physical, chemical, and cooking properties) of landraces without losing important information. The eigenvalues which measure the variation retained by the principal components were obtained to be 4.55, 2.61, 1.92, and 1.24 for the first four principal components. Also, the eigenvalues and variance percentage were evaluated from the scree plot (Figure 4) to determine the number of principal components to be considered. The first four components retained 73.8% of the variance where first, second, third, and fourth principal components (PC1, PC2, PC3, and PC4) depicted 32.5%, 18.7%, 13.7%, and 8.9% of the variance respectively. As seen in Figure 5, PC1 and PC2 were mostly related with the physical & chemical characteristics and elongation ratio of rice landraces. And PC3 and PC4 were mostly concerned with cooking variables (Figure 5). In PC1 (32.5%), the variables like the elongation ratio, milling recovery, hulling percentage, and grain breadth were positively correlated whereas the grain length and grain length-breadth ratio were negatively correlated. The second principal component PC2 (18.7%), showed positive correlation with gel consistency, grain breadth, bulk density, thousand kernel weight, and negative correlation with apparent amylose content. The third component PC3 (13.7%) was positively correlated with cooked length-breadth ratio and 1000 kernel weight but negatively with optimum cooking time. The fourth component PC4 (8.9%) depicted positive correlation with gruel solid loss. The biplot of individuals and variables in PC1 and PC2 (Figure 6) depicted that landraces like Lekali Marsi, Indrabeli, Juhari, Baryang Masino, and Thakali Lahare Marsi had high milling recovery, greater elongation ratio, and high apparent amylose content; landraces from the Anadi group like Pahelo Anadi, Anadi Tude, Anadi Local, and Seto Anadi had high gel consistency and thousand kernel weight but low apparent amylose content. Similarly, the biplot of individuals and variables in PC3 and PC4 (Figure 7) was analysed where landraces like Rato Masino and Jetho Budo had high gruel solid loss, landraces like Aanga, Pahele, Kalo Masino, Bihari, and Eakle had high cooking time, and landraces like Rato Anadi, Biramful, Jarneli, Chiniya, and Jhinuwa Local had high cooked length-breadth ratio.

## 4. Discussions

The milling characteristics are the important parameters to determine the grain quality for further processing of the milled rice as these are related with cooking and eating characters. In this study, we found landraces like Marsi, Baryang Masino, and Lekali Marsi have the highest hulling percentage which suggest they have a higher head rice recovery than long grain variety like Kalo Masino. Among the landraces not much variability was obtained in their milling characteristics. However, besides the varietal difference, the type of mill used for milling also highly affects the bran percentage and milling recovery [20].

The physical characteristics like grain dimensions and weight are of vital interest to those involved in many facets of the rice industry, and these are major criteria to judge rice grain quality. According to Thomas, Wan-Nadiah, and Bhat [4], physical properties are evaluated to provide important facts in determining their appropriate uses. Bulk density was inversely related to the *L* : *W* ratio, round grain showed the highest bulk density, and slender grain showed the lowest [21–23]. Similar findings were seen in this experiment, from the principle component analysis, we can conclude that bulk density and grain breadth are positively correlated and negatively correlated with length-breadth ratio. Also Pudke Dhan has the maximum bulk density and Kalo Masino with the minimum bulk density. This knowledge of bulk density is useful during storage and further processing. Another important physical characteristic that determines the rice cooking quality and preference is its grain size and shape. The rice length-breadth ratio is used to determine the shape of variety, and increase in length of rice variety is mostly preferred [11, 24]. Long-type landraces are still preferred by local people like Jetho Budo, Rato Masino, Chiniya, and Jarneli and even rice with medium-grain shape like Pahelo Anadi and Rato Anadi are preferred. According to Sharma et al. [6], these preferred landraces like Jetho Budo, Rato Anadi, and Pahelo Anadi also showed promising yield attributing characters with high grains per panicle and 1000 grain weight as well as greater filled grain percentage.

The chemical characters are said to be the determinants of cooking quality and are positively or negatively correlated with the cooking characteristics. The gelatinisation temperature depicts the cooking time required as it is highly related with cooking time [25]. It also affects the water uptake and kernel elongation [5]. They also reported that higher amylose content has less crystalline structure and has low gelatinisation temperature. According to Wu et al. [18], rice with high amylose content has an intermediate or low gelatinisation temperature whereas rice with low or waxy amylose content has a high or low gelatinisation temperature. Cuevas and Fitzgerald [26] reported that gelatinisation temperature as an indicator of cooking time and shorter cooking time are of great importance as it saves significant fuel amount. According to Oko, Ubi, and Dambaba [2], when cooked, rice types with hard gel consistency harden faster than those with a soft gel consistency. Rice with soft gel consistency cooks more tenderly and remains soft even after cooling. They also found the negative correlation between amylose content and gel consistency, suggesting unlikelihood of correlated responses in selecting for these traits. In this experiment also, PC2 showed an opposite relationship between gel consistency and apparent amylose content. Chemutai, Musyoki, and Kioko [27] explained gel consistency as the indirect method used in screening cooked rice for its hardness when cooked. Hard gel consistency is due to formation of rigid rice gels; as a result, association of starch polymers in the aqueous phase and intermediate gel consistency is due to the effects of minor genes, for instance gene interaction between waxy pullulanase or waxy and BEIII genes. Similarly, on this study, wide variation among the landraces was obtained for the gelatinisation behaviour. Among them, Pahelo Anadi, Anadi Local, and Seto Anadi possessed soft gelatinisation behaviour which means they remain moist and soft after cooking and this also explains the tenderness and sticky nature of these varieties. The preference of these varieties can also be further supported by high amounts of protein content and indication of longer cooking time too [5]. Thomas, Wan-Nadiah, and Bhat [4] stated in their experiment that amylose content plays a significant role in determining the overall cooking and eating quality of a rice variety, even though the quality is also affected by proteins, lipids, or amylopectin. Similarl finding was found in the experiment of Adu-Kwarteng et al. [24] and Asghar et al. [8]. Singh et al. [1] further stated that rice with intermediate amylose content of 20-25% are most preferred by consumers as they are reported to cook moist and remain soft (when cool). Also in this study, 19 of the landraces were found to be with intermediate amylose content and explains the preference of these landraces. High amylose content determines the quality of rice, pasting properties related to hardness and inversely related to stickiness [17]. This study supports the previous findings as Pahelo Anadi was found to be with low amylose content which is sticky in nature and Jetho Budo and Lekali Marsi was found with high amylose content which is dry and fluffy in nature after cooking. Wang, Wang, and Porter [28] reported that the difference in physicochemical characteristics of traditional rice varieties having similar range of amylose content may be due to the difference in branch length and chain length distribution of starch components. Also, when aroma was tested, some were found with strong and mild aroma which explains the further research for these aromatic traits to be used in breeding programmes.

Rice being a major staple food in most of the developing countries, cooking quality possesses utmost importance for its economy and marketing. Cooking time is also related to fuel and energy consumption, and longer cooking time may be due to the lesser degree of milling. Cooking time is also related to the amylose content and varied significantly among the varieties [5]. Hettiarachchi et al. [21] reported in their study that variation in the cooking time among different varieties could be related to the gelatinisation temperature; however, some varieties with a high gelatinisation temperature have shown low cooking time. In this study, landrace like Aanga has a high gelatinisation temperature which has shown the longest cooking time and Chiniya has shown the lowest cooking time and has a low gelatinisation temperature. However, some varieties have not followed this relation which may be due to lesser degree of milling. Vidal et al. [29] reported from their experiment that optimum cooking time is mainly linked with grain morphology of rice rather than its starch properties such as amylose content, and they do not find correlation between amylose content and cooking time. This study also showed similar results as optimum cooking time and apparent amylose content are in different principal components which are not related. Bhat and Riar [5] stated that the water uptake ratio is directly related to energy consumption during cooking and they found water uptake ratio to be higher for the local varieties than hybrid ones. They also showed a similar result in the relationship between the water uptake ratio and higher amylose content as well as bulk density. In this study, the highest water uptake ratio was found in Jetho Budo which has maximum amylose content and bulk density. Low solid loss is an attribute of slender rice grains having a comparatively smaller surface area; also, rice varieties with higher amylose content are prone to leach out solids into cooking water [21]. Highly polished rice is more vulnerable to gruel solid loss. Subedi et al. [30] reported that rice cooked immediately after harvest shows poor quality and is about to become pasty, fail to sell, more solids in solution, and fragmentation and these characters become progressively less with age. Thomas, Wan-Nadiah, and Bhat [4] in their study observed that the highest *l*/*b* ratio was obtained in white rice; they stated that breadthwise increase in cooking of rice is an undesirable trait, while high-quality rice varieties are characterized and preferred based on the increase in length. In this study also, the first principal component shows that landraces like Lekali Marsi, Thakali Lahare Marsi, Indrabeli, and Baryang Masino have a high elongation ratio which means they can fetch premium price in the market. A study on rice landraces from Lamjung and Tanahun districts reported wide diversity and huge variability for agromorphological characteristics of those landraces and suggested their utilisation in future breeding programme [31]. Thus, the result from this study in combination with similar other studies related to agromorphological characteristics can be a foundation for molecular study.

## 5. Conclusion

This study conducted on 30 landraces collected from Tanahun and Lamjung, revealed the variability of landraces based on their physicochemical and cooking characteristics. Landraces were highly significant in physicochemical characteristics as well as in cooking characteristics. However high variation among the landraces was only obtained in the characteristics like gel consistency, gruel solid loss, and water uptake ratio. Some of the landraces showed noble physicochemical and cooking characteristics that can fetch premium prices. Landraces belonging to the Anadi group showed high gel consistency and medium amylose content and landraces like Lekali Marsi, Thakali Lahare Marsi, Indrabeli, Baryang Masino, Jetho Budo, and Chiniya showed lengthwise elongation which gives them economy and market. The information obtained through this study could be used in rice breeding programmes and molecular research for further improvement of rice.

## Figures and Tables

**Table 1:** Gel consistency and cooking characteristics of rice landraces. Results are expressed as mean value ± SE. Mean in a column with the same small letters in subscript are not significantly different.

Landraces	GC (mm)	OCT (min)	WUR	GSL (%)	ER	CLBR
Aanga	5.87 ± 1.5^b_f^	44.4 ± 1.46^a^	6.05 ± 0.34^de^	1.79 ± 0.48^a_d^	1.34 ± 0.01^kl^	2.29 ± 0.03^mno^
Anadi Local	7.2 ± 1.15^a_d^	26.65 ± 1.16^ij^	5.94 ± 0.13^de^	0.99 ± 0.28^b_f^	1.37 ± 0.01^jk^	2.44 ± 0.03^klm^
Anadi Tude	9.2 ± 0.3^a^	29.92 ± 1.18*gh*	4.99 ± 0.09*f*^_k^	0.62 ± 0.14^def^	1.51 ± 0.03*ghi*	2.65 ± 0.09^ijk^
Baryang Masino	6.1 ± 0.2^b_e^	33.69 ± 0.28^f^	5.1 ± 0.09^f_j^	0.69 ± 0.35^def^	1.66 ± 0.02^def^	2.9 ± 0.11^ghi^
Bihari	5.1 ± 1.77^d_i^	36.64 ± 2.31^cde^	4.8 ± 0.17^g_l^	1.37 ± 0.31^b_e^	1.4 ± 0.06^ijk^	2.64 ± 0.09^ijk^
Biramful	2.73 ± 0.62^h_k^	24.49 ± 0.28^jkl^	7.52 ± 0.33^ab^	1.56 ± 0.28^b_e^	1.38 ± 0.03^jk^	2.96 ± 0.09^fgh^
Chiniya	4.37 ± 0.84^d_j^	23.45 ± 0.02^l^	4.71 ± 0.04^h_l^	0.1 ± 0.00^f^	1.55 ± 0.01^fgh^	2.81 ± 0.02^hij^
Eakle	6.03 ± 0.31^b_e^	37.45 ± 0.05^c^	5.03 ± 0.16^f_k^	1.83 ± 0.46^a_d^	1.79 ± 0.04^c^	2.81 ± 0.08^hij^
Gokule Mansuli	2.43 ± 1.03^ijk^	36.88 ± 1.14^cd^	5.61 ± 0.28^ef^	0.89 ± 0.26^c_f^	1.76 ± 0.04^cd^	3.01 ± 0.15^fgh^
Indrabeli	2.03 ± 0.53^jk^	26.24 ± 0.95^i_l^	4.57 ± 0.14^i_m^	1.01 ± 0.5^b_f^	1.92 ± 0.02^b^	3.18 ± 0.08^def^
Jarneli	4.4 ± 0.4^d_j^	23.85 ± 0.26^kl^	6.62 ± 0.17^cd^	0.87 ± 0.1^c_f^	1.39 ± 0.05^jk^	2.61 ± 0.11^jkl^
Jetho Budo	5.57 ± 1.06^b_h^	25.59 ± 1.36^i_l^	7.96 ± 0.03^a^	2.13 ± 0.14^ab^	1.56 ± 0.01^fgh^	3.78 ± 0.09^ab^
Jhinuwa Local	2.87 ± 0.73^f_k^	25.83 ± 0.4^i_l^	5.12 ± 0.01^f_j^	0.35 ± 0.05^ef^	1.7 ± 0.04^cde^	3.42 ± 0.17^cd^
Juhari	1.67 ± 0.32^jk^	33.3 ± 1.37^f^	4.97 ± 0.14^f_k^	1.08 ± 0.14^b_f^	1.94 ± 0.04^b^	2.96 ± 0.15^fgh^
Kalo Jhinuwa	3.2 ± 1.4^e_k^	32.35 ± 0.22^fg^	4.78 ± 0.14^g_l^	1.5 ± 0.05^b_e^	1.31 ± 0.05^klm^	2.34 ± 0.05^lmn^
Kalo Masino	1.27 ± 0.12^k^	37.19 ± 0.04^c^	4.48 ± 0.38^i_m^	1.94 ± 0.23^abc^	1.65 ± 0.03^ef^	2.86 ± 0.09^g_j^
Kalo Namdunge	2.83 ± 1.83^g_k^	28.34 ± 0.44^hi^	4.13 ± 0.08^lm^	0.77 ± 0.16^c_f^	1.94 ± 0.00^b^	3.07 ± 0.17^fgh^
Kathe	7.1 ± 1.55^a_d^	39.33 ± 0.04^bc^	5.58 ± 0.07^ef^	0.45 ± 0.29^ef^	1.32 ± 0.03^kl^	2.05 ± 0.05^o^
Lekali Marsi	3.47 ± 0.85^e_k^	27.05 ± 1.77^ij^	5.45 ± 0.16^efg^	1.11 ± 0.05^b_f^	1.98 ± 0.02^b^	3.57 ± 0.06^bc^
Mansara	3.53 ± 1.15^e_k^	38.17 ± 0.03^c^	6.88 ± 1.05^bc^	1.75 ± 0.07^a_d^	1.6 ± 0.03^efg^	3.04 ± 0.15^fgh^
Marsi	4.07 ± 0.86^e_k^	38.08 ± 0.66^c^	4.75 ± 0.15^g_l^	1.44 ± 0.21^b_e^	1.65 ± 0.01^def^	2.59 ± 0.07^jkl^
Pahele	8.4 ± 2.63^ab^	41.41 ± 1.89^b^	4.4 ± 0.07^j_m^	0.75 ± 0.06^c_f^	1.47 ± 0.06^hij^	2.64 ± 0.06^ijk^
Pahelo Anadi	9.73 ± 0.54^a^	25.37 ± 0.1^jkl^	5.69 ± 0.35^ef^	1.41 ± 0.24^b_e^	1.93 ± 0.05^b^	3.4 ± 0.06^cde^
Pakhe Sali	5.8 ± 0.4^b_g^	32.62 ± 2.4^fg^	5.38 ± 0.01^e_h^	0.75 ± 0.1^c_f^	1.23 ± 0.02^lm^	2.07 ± 0.06^no^
Pudke Dhan	3.57 ± 0.97^e_k^	34.28 ± 0.09^def^	4.4 ± 0.07^j_m^	1.04 ± 0.25^b_f^	1.95 ± 0.02^b^	2.86 ± 0.04^g_j^
Rato Anadi	5.13 ± 0.8^c_i^	26.37 ± 1.0^ijk^	3.91 ± 0.03^m^	0.38 ± 0.08^ef^	1.7 ± 0.02^cde^	3.11 ± 0.09^fg^
Rato Masino	8.13 ± 0.63^abc^	26.29 ± 0.12^ijk^	4.32 ± 0.08^klm^	2.84 ± 1.6^a^	1.21 ± 0.08^m^	2.51 ± 0.03^klm^
Seto Anadi	9.27 ± 0.73^a^	31.87 ± 0.28^fg^	5.14 ± 0.05^f_i^	1.25 ± 1.0^b_e^	1.63 ± 0.02^ef^	3.13 ± 0.19^efg^
Sobhara	4.27 ± 1.11^d_k^	33.96 ± 0.25^ef^	5.43 ± 0.18^e_h^	1.82 ± 0.08^a_d^	1.68 ± 0.07^cde^	3.05 ± 0.06^fgh^
Thakali Lahare Marsi	2.33 ± 0.83^ijk^	37.57 ± 0.27^c^	5.36 ± 0.16^e_h^	0.74 ± 0.17^c_f^	2.41 ± 0.05^a^	3.87 ± 0.06^a^
*p* value	5.19*e*^−08^	<2*e*^−16^	<2*e*^−16^	0.0091	<2*e*^−16^	<2*e*^−16^
Sig.	<0.001(^∗∗∗^)	<0.001(^∗∗∗^)	<0.001(^∗∗∗^)	<0.01(^∗∗^)	<0.001(^∗∗∗^)	<0.001(^∗∗∗^)
LSD	3.003	2.79	0.737	1.20	2.00	0.28
CV (%)	37.36	5.35	8.52	63.06	4.15	5.97

Note: a_d: abcd.

## Data Availability

The data used to support the findings of this study are available from the corresponding author upon request.

## Supplementary Materials

Supplementary materials The supplementary material contains a table representing milling (hulling percentage and milling recovery) and physical characteristics (bulk density and 1000 kernel weight) of evaluated 30 rice landraces.
