# Supplementary material for: Evaluation of Physicochemical and Cooking Characteristics of Rice (Oryza sativa L.) Landraces of Lamjung and Tanahun Districts, Nepal
Source: Int J Food Sci. 2020 Aug 18;2020:1589150. doi: 10.1155/2020/1589150 (PMC7450303; doi:10.1155/2020/1589150)
Supplement: Supplementary materials — The supplementary material contains a table representing milling (hulling percentage and milling recovery) and physical characteristics (bulk density and 1000 kernel weight) of evaluated 30 rice landraces. [file 1589150.f1.docx]

Table 2: Milling characteristics and Physical characteristics of evaluated rice landraces

| **Landraces** | **Hulling Percentage (%)** | **Milling Recovery (%)** | **Bulk Density (g/cm^3^)** | **1000 Kernel Weight (g)** |
| --- | --- | --- | --- | --- |
| Pahelo Anadi | 76.18 | 68.26 | 0.86 | 21.18 |
| Rato Anadi | 77.41 | 70.46 | 0.87 | 23.19 |
| Gokule Mansuli | 78.44 | 73.11 | 0.86 | 14.68 |
| Rato Masino | 78.26 | 71.03 | 0.84 | 25.65 |
| Thakali Lahare Marsi | 80.09 | 72.69 | 0.88 | 19.54 |
| Pudke Dhan | 79.96 | 74.19 | 0.88 | 18.97 |
| Eakle | 78.58 | 72.34 | 0.87 | 15.11 |
| Kalo Masino | 76.19 | 66.98 | 0.81 | 12.62 |
| Biramful | 78.43 | 69.20 | 0.84 | 16.90 |
| Indrabeli | 81.03 | 75.55 | 0.83 | 19.24 |
| Kalo Namdunge | 80.76 | 74.65 | 0.87 | 20.58 |
| Jarneli | 77.22 | 68.33 | 0.86 | 19.98 |
| Aanga | 77.14 | 67.86 | 0.87 | 19.98 |
| Jetho Budo | 78.79 | 70.09 | 0.82 | 21.32 |
| Kalo Jhinuwa | 78.20 | 72.62 | 0.84 | 13.86 |
| Jhinuwa Local | 78.64 | 71.57 | 0.86 | 13.88 |
| Anadi Tude | 76.90 | 69.75 | 0.87 | 22.26 |
| Lekali Marsi | 81.67 | 74.11 | 0.84 | 21.03 |
| Baryang Masino | 81.72 | 74.93 | 0.84 | 15.79 |
| Sobhara | 78.32 | 71.19 | 0.87 | 17.80 |
| Marsi | 82.53 | 73.72 | 0.84 | 20.40 |
| Anadi Local | 76.99 | 69.46 | 0.84 | 22.59 |
| Pakhe Sali | 77.17 | 69.74 | 0.83 | 17.87 |
| Mansara | 78.05 | 69.39 | 0.81 | 17.36 |
| Pahele | 77.03 | 70.78 | 0.84 | 16.99 |
| Chiniya | 77.22 | 68.32 | 0.85 | 21.06 |
| Juhari | 79.84 | 70.63 | 0.85 | 16.46 |
| Seto Anadi | 77.37 | 69.35 | 0.87 | 21.93 |
| Kathe | 76.76 | 68.01 | 0.87 | 17.37 |
| Bihari | 78.25 | 71.30 | 0.84 | 17.54 |
| **Mean** | **78.50** | **70.99** | **0.85** | **18.77** |
| **SD** | **1.71** | **2.36** | **0.02** | **3.11** |
| **CV(%)** | **2.18** | **3.32** | **2.34** | **16.55** |
